# Performance study of a 360° CZT camera for monitoring ^177^Lu-PSMA treatment

**DOI:** 10.1186/s40658-023-00576-1

**Published:** 2023-09-22

**Authors:** Laure Vergnaud, Jean-Noël Badel, Anne-Laure Giraudet, David Kryza, Thomas Mognetti, Thomas Baudier, Hanan Rida, Arnaud Dieudonné, David Sarrut

**Affiliations:** 1 Centre de lutte contre le cancer Léon Bérard, Lyon, France; 2 CREATIS, CNRS UMR 5220, INSERM U 1044, Université de Lyon, INSA-Lyon, Université Lyon 1, Lyon, France; 3 Hospices Civils de Lyon, Université de Lyon, Université Claude Bernard Lyon 1, LAGEPP UMR 5007 CNRS, Lyon, France; 4 Département de médecine nucléaire, Centre Henri Becquerel, Rouen, France

**Keywords:** CZT camera, SPECT, ^177^Lu, Theranostic, Internal radiotherapy

## Abstract

**Background:**

The aim of this study was to investigate the quantification performance of a 360° CZT camera for ^177^Lu-based treatment monitoring.

**Methods:**

Three phantoms with known ^177^Lu activity concentrations were acquired: (1) a uniform cylindrical phantom for calibration, (2) a NEMA IEC body phantom for analysis of different-sized spheres to optimise quantification parameters and (3) a phantom containing two large vials simulating organs at risk for tests. Four sets of reconstruction parameters were tested: (1) Scatter, (2) Scatter and Point Spread Function Recovery (PSFR), (3) PSFR only and (4) Penalised likelihood option and Scatter, varying the number of updates (iterations × subsets) with CT-based attenuation correction only. For each, activity concentration (ARC) and contrast recovery coefficients (CRC) were estimated as well as root mean square. Visualisation and quantification parameters were applied to reconstructed patient image data.

**Results:**

Optimised quantification parameters were determined to be: CT-based attenuation correction, scatter correction, 12 iterations, 8 subsets and no filter. ARC, CRC and RMS results were dependant on the methodology used for calculations. Two different reconstruction parameters were recommended for visualisation and for quantification. 3D whole-body SPECT images were acquired and reconstructed for ^177^Lu-PSMA patients in 2–3 times faster than the time taken for a conventional gamma camera.

**Conclusion:**

Quantification of whole-body 3D images of patients treated with ^177^Lu-PSMA is feasible and an optimised set of parameters has been determined. This camera greatly reduces procedure time for whole-body SPECT.

## Background

Radiopharmaceutical therapy (RPT) targeting the prostate-specific membrane antigen (PSMA) [1], labelled with lutetium-177 (^177^Lu-PSMA) in patients with metastatic castration-resistant prostate cancer (mCRPC), has shown remarkable responses in several clinical studies [2–6] and is now clinically proposed to eligible patients in an increasing number of nuclear medicine departments worldwide. However, the therapeutic window, i.e. the response to treatment without adverse effects, depends on several factors including patient-specific organ function, tracer pharmacokinetics and tumour uptake, implying the need for treatment monitoring.

The European Association of Nuclear Medicine (EANM) has recently provided general guidelines to ensure good practice standards to follow for this treatment [7]. In particular, the implementation of individualised dosimetry according to the European Directive 2013/59/Euratom is mentioned. This stage involves calculating the absorbed dose in tumours and healthy organs for each treated patient.

SPECT imaging is currently the only source of information for estimating absorbed dose after a patient’s therapeutic administration. Quantification can be performed with 2D planar scintigraphy [8–10] or from 3D reconstructed images [11, 12], the latter showing better accuracy [13]. More details about absorbed dose estimation from 3D SPECT images may be found in [14, 15].

However, SPECT/CT acquisition is time-consuming. With a conventional Anger gamma camera, a whole-body SPECT examination (about 5 × 40 cm bed positions (BP) for a patient measuring 170–180 cm) takes around 12 to 30 min per BP, therefore around 60 to 150 min overall, depending upon the acquisition parameters [16–19]. Such long acquisition times are problematic for patients in pain and have consequences on the availability of medical staff and camera scheduling. Thus, scan acquisition time has been an impediment to routine clinical implementation of ^177^Lu treatment monitoring.

More recently, cadmium–zinc–telluride (CZT) detector SPECT/CT cameras have been available. The advantages of direct digital conversion with CZT detectors compared to conventional Anger-based analogue technology have been reported [20–23]. Instead of converting incident gamma photons into visible light photons and then into electrical signals using photomultiplier tubes, CZT technology enables direct photon energy conversion, thus improving detection efficiency, energy resolution and spatial resolution, and therefore enabling reduced acquisition times. However, Rit et al. [22] recently reminded that these enhancements cannot be attributed to CZT detectors alone, but are also due to additional improvements, including adapted collimation and pixelated detectors.

Among the commercially available CZT-based SPECT and SPECT/CT systems (D-SPECT and Discovery NM 530c for myocardial applications; Discovery NM/CT 670 or 870 CZT and StarGuide ([24]) for other applications), the VERITON-CT camera (Spectrum Dynamics Medical) is composed of twelve mobile CZT detector heads covering 360°. Detectors can be rotated and moved independently to be as close as possible to the patient’s surface contour to increase the solid angle and thus improve photon collection efficiency, as described by Goshen et al. [25]. These features enable significant time reductions for whole-body SPECT image acquisition compared to conventional gamma cameras. Consequently, it facilitates the opportunity for clinical implementation of ^177^Lu treatment dosimetry. Recent studies evaluating VERITON performances have been conducted for various clinical procedures with ^99m^Tc radionuclides. Indeed, Bordonne et al.[26] compared ^99m^Tc-HMPAO brain perfusion SPECT using this system and a conventional Anger camera (Symbia T2, Siemens Healthineers). They found twofold higher sensitivity for the CZT camera and enhancement of grey/white matter contrast. Imbert et al.[27] investigated ^99m^Tc-Sestamibi myocardial perfusion in morbidly obese patients and concluded that it can replicate the characteristics of a dedicated cardiac CZT camera, thus enabling easier management of severely obese patients. Desmonts et al.[28] evaluated the CZT camera performance by comparison with a conventional dual-head Anger camera (dual-head Symbia, Siemens Healthineers) for several radioelements (^99m^Tc, ^123^I, ^201^Tl, ^111^In). They found the CZT camera had an energy resolution, depending on radioisotope, ranging between 1.68 and 2.55 times higher than Anger camera. The sensitivity for a point source placed in air was between 1.6 and 8 times higher for the CZT camera compared with the Anger camera, depending on whether the focus mode was activated, which reduces the swipe motion of detectors to a user-defined region of interest.

The CZT detection system for the VERITON-CT 200 series camera used in this study has a SPECT energy range of 40–200 keV, therefore preventing the acquisition of the higher ^177^Lu photopeak (208 keV, 10.4%), thus restricting acquisition to the lower photopeak (113 keV, 6.2%) and affected by additional scattered photons of higher energy. To our knowledge, only one case report has been published for this system in association with ^177^Lu treatment of neuroendocrine tumours [29]. Another study was conducted on the CZT StarGuide gamma camera to determine if it enables faster post-therapy whole-body SPECT/CT acquisitions (^177^Lu-DOTATATE and ^177^Lu-PSMA) compared to a conventional camera, while maintaining equal or higher detection rates. This study did not focus on the quantitative aspects of the acquired images [24]. Finally, Kennedy et al.[30] have evaluated the accuracy of activity concentration measurements for ^177^Lu therapy using the Discovery 670 CZT camera (conventional camera geometry). They computed recovery coefficients for a NEMA IEC phantom and compared the estimated activity concentration in the bladder (images) with the actual activity concentration in the urinary of patients undergoing ^177^Lu-PSMA treatment.

In this work, we studied performance of the VERITON-CT camera for ^177^Lu imaging with various phantoms and characterised the current absolute quantification capabilities of the system for monitoring and dosimetry of ^177^Lu-PSMA treatments.

## Material and methods

### SPECT imaging system

This study was performed with the VERITON-CT (Spectrum Dynamics, Caesarea, Israel) hybrid CZT camera installed at the nuclear medicine department of the Léon Bérard Center (Lyon, France). The system is composed of twelve CZT detector columns regularly spaced around 360°, that swivel to acquire data from the entire field of view of 32 cm in axial direction, as detailed in [28]. Each detector module consists of 128 × 16 pixel solid-state detectors with non-removable tungsten parallel hole collimators. This system detects photons in the energy range 40–200 keV, which limits the use of certain isotopes and/or photopeaks (e.g. 208 keV peak of ^177^Lu).

### Phantom experiments

**Fig. 1:**
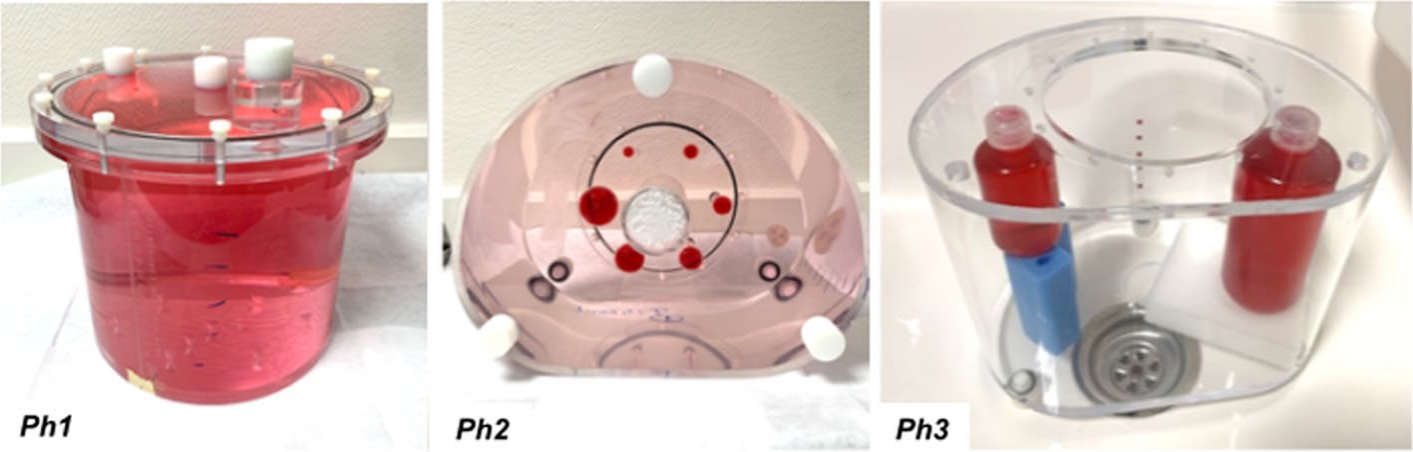
Phantoms used for ^177^Lu quantification performances evaluation. From left to right: uniform water cylinder (Ph1), NEMA IEC Body (Ph2) and NEMA IEC Body modified with two internal hot vials (Ph3). The red colour indicates the ^177^Lu radioactive volumes in each phantom

Quantification performances were investigated using three different phantoms: a uniform water cylinder (Ph1), a standard NEMA IEC Body phantom (Ph2) and a NEMA IEC Body phantom modified with two internal hot vials (Ph3) (Fig. 1). Table 1 gives detailed information for these three phantoms. The phantoms were filled with different activity concentrations of ^177^Lu as indicated in Table 1 (activities shown are those at first time-point acquisition). Ph1 phantom was used for calibration, Ph2 was used for conventional analysis of different-sized spheres, and Ph3 contained larger hot volumes, surrounded by water, simulating two “organs” (129 and 521 mL). Acquisitions were performed with a ratio of 7:1 for Ph2 like Santoro et al. [17]. Note that other ratios were also used from 2.7:1 to 13:1 [31–35].

**Table 1:** Description of the phantoms used for this study. The activity values are given at the first acquisition time of each phantom

	*Ph1*	*Ph2*	*Ph3*
Phantom description	Uniform water cylinder	NEMA IEC Body	Modified NEMA IEC with hot vials
Usage	Calibration	ARC and CRC calculations	Large volumes testing
Radioactive background volume (mL)	6805	9658	NA
Insert volume (mL)	None	100 (stock solution)	Vial 1 = 130 Vial 2 = 535
*Acquisition session*			
Activity in background (MBq)	814	783	None
Activity in inserts (MBq) (or in stock solution)	None	58	Vial 1 = 129 Vial 2 = 521

*NA* not applicable

### Image acquisitions

All SPECT images were acquired with 4 angular acquisition steps (or orbits to cover the entire field of view) around the phantom, and one single-bed position. During each orbit, the detector columns swept through 60 angular projections of 3.75 s each in step-and-shoot mode, giving a total data acquisition duration of 900 s. The energy windows used for photopeak and scatter are described in Table 2. Acquisitions were performed for all three phantoms, at 8 timepoints, from 4 to 339 h after injection, as shown Table 3.

**Table 2:** Initial reconstruction parameters used for quantification and visualisation

	*RecQuant*	*RecVis*
Reconstruction algorithm	OSEM	OSEM
(Software)	VERITON 2.3.0.1234	VERITON 2.3.0.1234
Iterations	4	4
Subsets	8	8
Attenuation correction	CT-based	CT-based
Scatter correction	Yes	No
Main Photopeak window (keV)	[101.6; 124.2]	[101.6; 124.2]
Peak scatter windows at 113 keV	[79; 101.6] [124.2; 146.8]	[79; 101.6] [124.2; 146.8]
PSFR	No	PSFRd
HPC	No	Yes (HPC = 0.4)
Intra-filter	No	Convolution (pr:0.125, freq: 2)
Post-filter	No	Median (p: 3)
In-plane size	256 × 256	256 × 256
Pixel size	2.46^2^ mm^2^	2.46^2^ mm^2^

**Table 3:** SPECT/CT image acquisitions performed for Ph1, Ph2 and Ph3 at different timepoints after phantom injection of the first acquisition session. CT acquisitions with 17 effective mAs

No. acquisition	*Ph1*	*Ph2*	*Ph3*
1	5.1 h	4 h	3.5 h
2	27.6 h	25.3 h	18.5 h
3	43.5 h	42.3 h	43.1 h
4	72.4 h	74.4 h	NA
5	187.9 h	186.5 h	170.5 h
6	194.5 h	194.9 h	NA
7	241.4 h	241 h	218.9 h
8	338.6 h	338.4 h	NA

*NA* not acquired

Low-dose CT acquisitions were performed for attenuation correction. A voltage of 120 kVp and a current/time product of 17 mAs effective (mAs effective = mAs/pitch factor) were used to generate the X-ray beam. The CT images had slice thicknesses of 2.5 mm in the cranio-caudal direction and less than 1 mm in the other two directions.

### Reconstruction parameters

Two reconstruction protocols were investigated, the first for quantification and dosimetry purposes, denoted RecQuant, and the second one for visualisation assessment, denoted RecVis.

The initial parameters used for RecQuant and RecVis are given in Table 2. In a whole-body acquisition, the final volumes were composed of around 700–800 slices. Attenuation correction was performed using the CT image. Scatter correction was based on the equation described in [33]: *C*_prim_ = *C*_total_ − (*k*_1_ − *k*_2_ × *k*_3_)*C*_ds_ − *k*_2_ × *C*_s_, where *C*_prim_, *C*_total_, *C*_ds_ and *C*_s_ are the absolute number for counts of unscattered, total, downscatter and scatter photons, respectively. Here, *k*_1_ = *k*_2_ = 0.5 and *k*_3_ = 0. No scatter correction was used for RecVis as this is partially performed within the high peak correction (HPC) option, which is used to correct for high-energy photons passing through the collimator (parameter: 0.4 by default; parameter ∈ [0; 10[). Energy windows were the same for both protocols. Point Spread Function Recovery display (PSFRd) models the detector–collimator response and corrects for blurring effects (scatter); this was optimised for visualisation only. For RecVis, two additional filters were used: a convolution filter was applied during reconstruction and a median filter post-reconstruction.

### Camera calibration

**Fig. 2:**
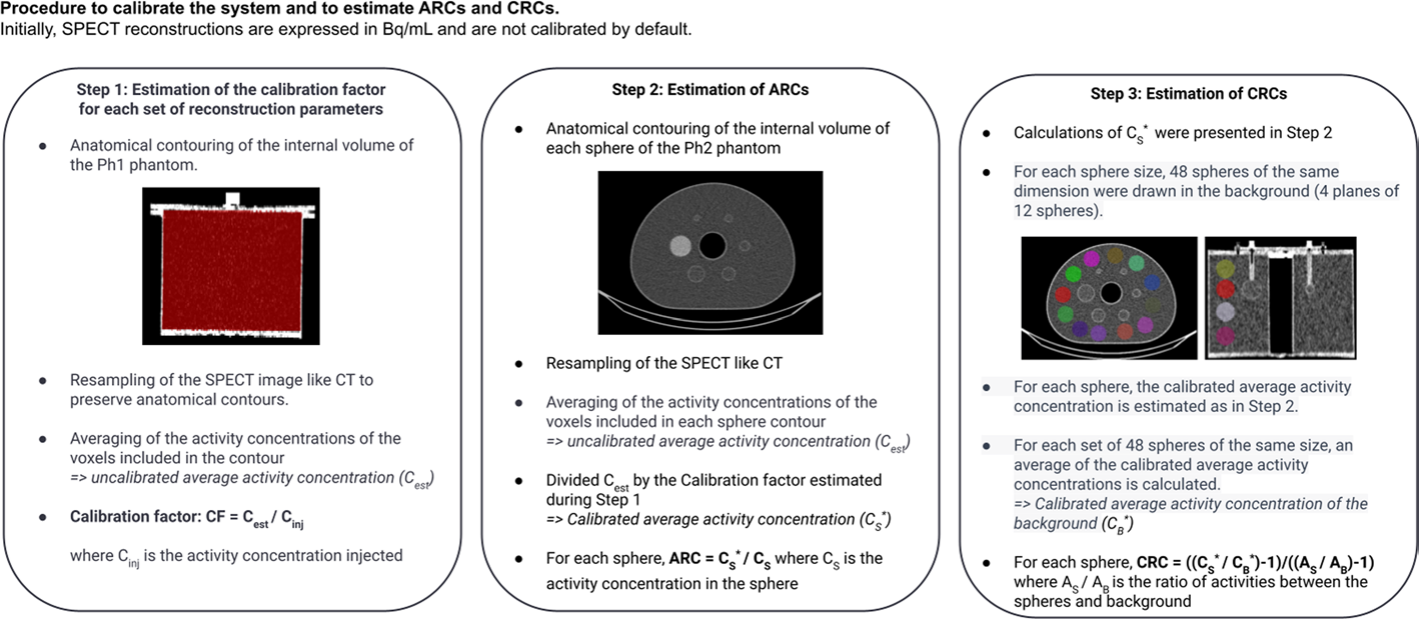
Schematic diagram showing the steps involved in estimating the calibration factors, ARCs and CRCs

The calibration factor (CF) was used to convert the number of detected counts into activity concentration in Bq/mL. We followed the MIRD pamphlet n^o^26 [14] and acquired a SPECT image of a large uniform hot water cylinder (Ph1 phantom here), where the total injected activity was assumed to be entirely within the phantom. The CF could be computed using ${{CF}} = \frac{{C_{est} [Bq/mL]}}{{C_{inj} [Bq/mL]}}$ where *C*_inj_ is the actual injected activity concentration in the phantom and *C*_est_ is the estimated activity concentration inside the phantom. To do this, the activity concentrations of the voxels within the phantom are averaged and then multiplied by its volume. The CF is therefore dimensionless. The number of reconstructed counts taking into account the corrections is not accessible, which does not allow estimating the calibration factor in counts/s/MBq as usually done in the literature. More details are available in Fig. 2.

Ph1 acquisition was repeated at different timepoints after injection corresponding to ^177^Lu radioactive concentrations ranging from 122.1 ± 0.5 kBq/mL to 28.7 ± 0.2. All prepared activities of ^177^Lu were measured using a Lemerpax scintiDOSE dose calibrator initially calibrated with a calibration vial of ^177^Lu provided by Advanced Accelerator Applications (Saint Genis Pouilly, France). Quality control of the sensitivity was performed before each acquisition time using the dedicated ^57^Co calibration source according to the manufacturer’s recommendations. Note that CF depends on the reconstruction parameters and was computed for all different reconstructions, as described in the next section.

### Evaluation criteria

To optimise the reconstruction parameters for quantification, the activity concentration recovery coefficients ARC [36] (for quantification), the contrast recovery coefficients CRC (for visual detection) and the variability in spheres and background (noise estimation) were computed on the Ph2 phantom as shown in Fig. 1. The mean ARCs were computed with Eq. (1), given in the MIRD pamphlet no. 23 [37], with C^*^_S_, the estimated activity concentration in the ^177^Lu sphere and C_S_, actual activity concentration in the sphere. Activity in the sphere was estimated by averaging activity concentrations in the sphere multiplied by the volume of the sphere. A CF estimated for each set of acquisition/reconstruction parameters was then applied. Spheres were delineated on the CT scan: therefore calculated volumes were close but not strictly equal to actual volumes, which explains why activities have been replaced by activity concentrations in Eq. (1). Differences between calculated and actual volumes were less than 9.5% (6.1 mL calculated vs 5.6 mL actual) except for the smallest sphere volume with a difference of 31% (0.36 mL calculated vs 0.52 mL actual).

1 $${{ARC}} = \frac{{C_{S}^{*} }}{{C_{S} }}$$

The contrast recovery coefficients (CRC) were computed as proposed in NEMA 2007. In Eq. (2), *C*_*S*_^∗^ was the estimated activity concentration in the^177^Lu sphere and *C* _*B*_^∗^ the estimated background activity concentration. $\frac{A_{S}}{A_{B}}$was the ratio of activity between the spheres and background. To compute *C* _*B*_^∗^, 48 spheres, with the same size as the ^177^Lu sphere, were segmented into four sections of the phantom in the cranio-caudal direction (with 12 3D spheres per section) of the CT scan. Activities were computed in all spheres, as was done for the ^177^Lu sphere, and then averaged. Note that activity of the background was computed for all spheres of different sizes. The variability in spheres and background was computed with the RMS formula (root mean square) as proposed by Ramonaheng et al.[38] or found in NEMA 2007. For the background, the contours used were those of the 48 segmented spheres for each sphere volume. More details about ARCs and CRCs are available in Fig. 2.

2 $${{CRC}} = \frac{{\frac{{C_{S}^{*} }}{{C_{B}^{*} }} - 1}}{{\frac{{A_{S} }}{{A_{B} }} - 1}}$$

3 $${{RMS}}(\% ) = \frac{SD}{{Mean}} \times 100$$

### Optimisation and evaluation of reconstruction parameters

All reconstructions were performed with OSEM, CT-based attenuation correction and geometry modelling. In addition, four sets of additional parameters were tested for various updates (iterations × subsets) as shown in Table 4. For set no. 1, scatter correction was added. For set no. 2, penalised likelihood (PL) regularisation was added in addition to scatter correction. The PL implementation of the manufacturer is inspired by [39, 40] and allows an increase in the number of iterations without much increase in noise. The penalty strength can be adjusted with two parameters: a regularisation parameter β that controls “the noise-resolution trade-off” and the frequency (freq). Default values are β = 1 and freq = 2. For sets no. 3 and no. 4, PSFRq (Point Spread Function Recovery optimised for quantification) option was used. PSFRq includes conventional, spatially invariant PSF together with blur corrections (scatter). Test no. 3 was with and test no. 4 was without additional scatter correction. No PL was applied for no. 3 and no. 4. Note that a specific calibration factor was computed for each set of reconstruction parameters. In addition, ARC was also estimated for the Ph3 phantom.

**Table 4:** Reconstruction parameters associated with each set whose number of updates varies between 32 and 384

	Set no. 1	Set no. 2	Set no. 3	Set no. 4
Reconstruction algorithm	OSEM	OSEM	OSEM	OSEM
Regularisation	No	Yes (PL)	No	No
Attenuation correction	CT-based	CT-based	CT-based	CT-based
Scatter correction	Yes	Yes	Yes	No
PSFRq option	No	No	Yes	Yes

### Impact of the segmentation

To our knowledge, there is no consensus in the literature on how to determine the ROI used to compute ARC and CRC. Some authors used contours from the CT image (M1) [41, 42], others reduced those contours to obtain smaller spheres inside the “physical” spheres (M2) [43, 44]. Other authors used thresholding on the SPECT image (M3) [32] as in Eq. 4 where AC_thresh_, AC_Max_(Sph) and AC_Mean_(bg) correspond to the threshold estimated for one sphere, the maximum activity concentration in the sphere and the mean activity concentration in the background. The ARCs were estimated from the mean and maximum activity concentration as proposed by Peters et al. [45]. We compared the different approaches.

4 $$AC_{thresh} = \frac{1}{2} \times (AC_{Max} (Sph) + AC_{Mean} (bg))$$

### Patient images

The estimated count rates in the phantoms (in particular Ph2) were compared to the estimated count rates from each BP (6 or 7 total) for three patients (administered activities: 6027 MBq, 6259 MBq and 7309 MBq) at three different timepoints (5–6.1 h, 23.1–25.7 h and 142.1–144.1 h) to evaluate whether count rates in the phantoms are representative of those in patients. The number of counts detected in the projections as well as the acquisition time for each BP could be obtained using the time reduction application (TR) on the gamma camera workstation. For illustration purposes, ^68^ Ga-PSMA PET and ^177^Lu-PSMA-1 SPECT images of a patient treated for metastatic castration-resistant prostate cancer are given. A SPECT image was acquired 24 h after each therapeutic administration (5 injections in total). For this patient, the acquisition time after cycle 1 was 24 min. Images were reconstructed with RecVis and projected (MVP “multi-view planar” option) to obtain a planar image. For the first therapy, the 24 h SPECT image was also reconstructed with RecQuant (12 iterations and 8 subsets). Acquisition duration was reported.

## Results

### Iterations and subsets

**Fig. 3:**
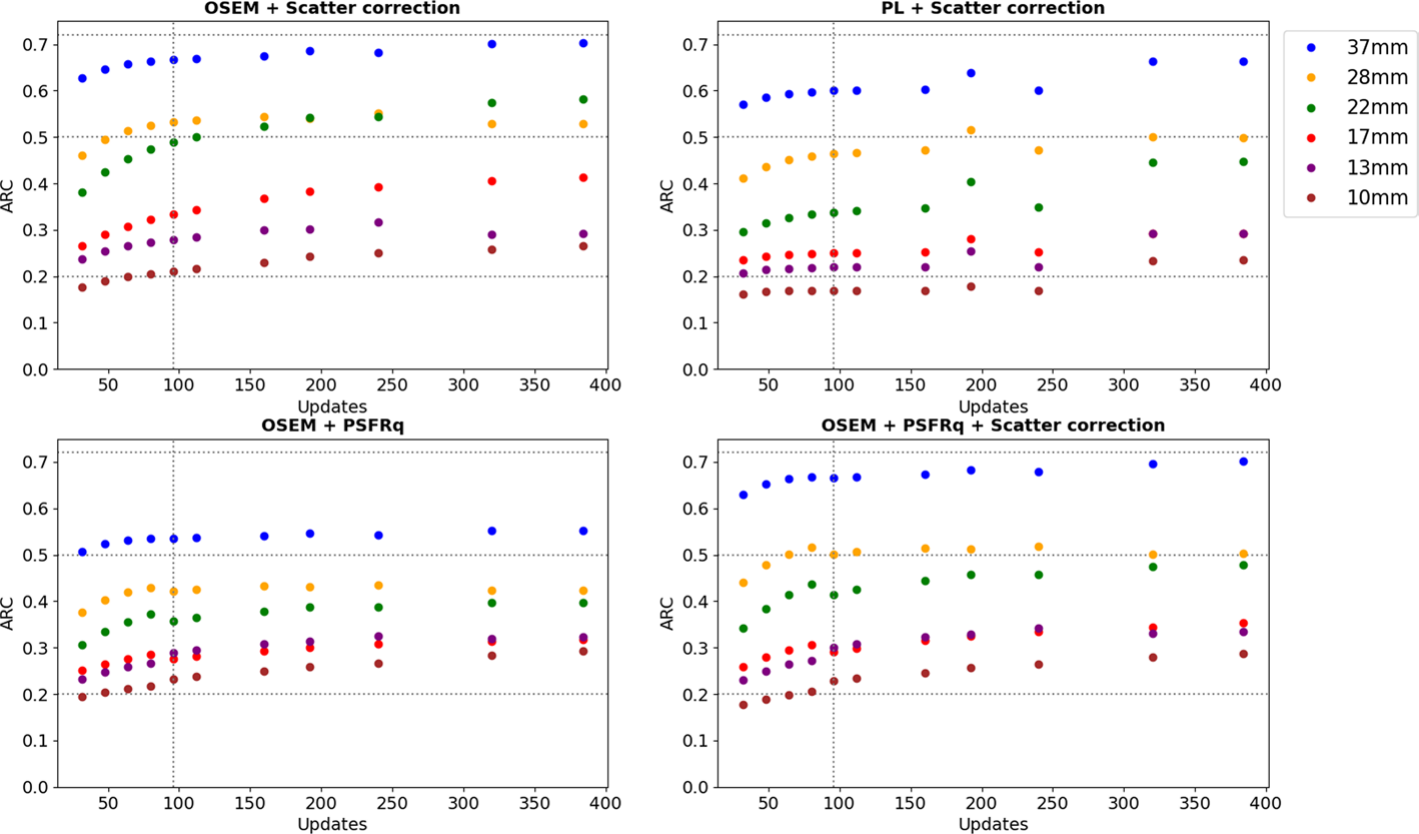
Activity concentration recovery coefficients (ARC) for different volumes of spheres as a function of the number of updates. At the top left, a scatter correction is applied. Top right, a PL reconstruction is used as well as a scatter correction. Below, the PSFRq option is applied with (right) or without scatter correction (left)

**Fig. 4:**
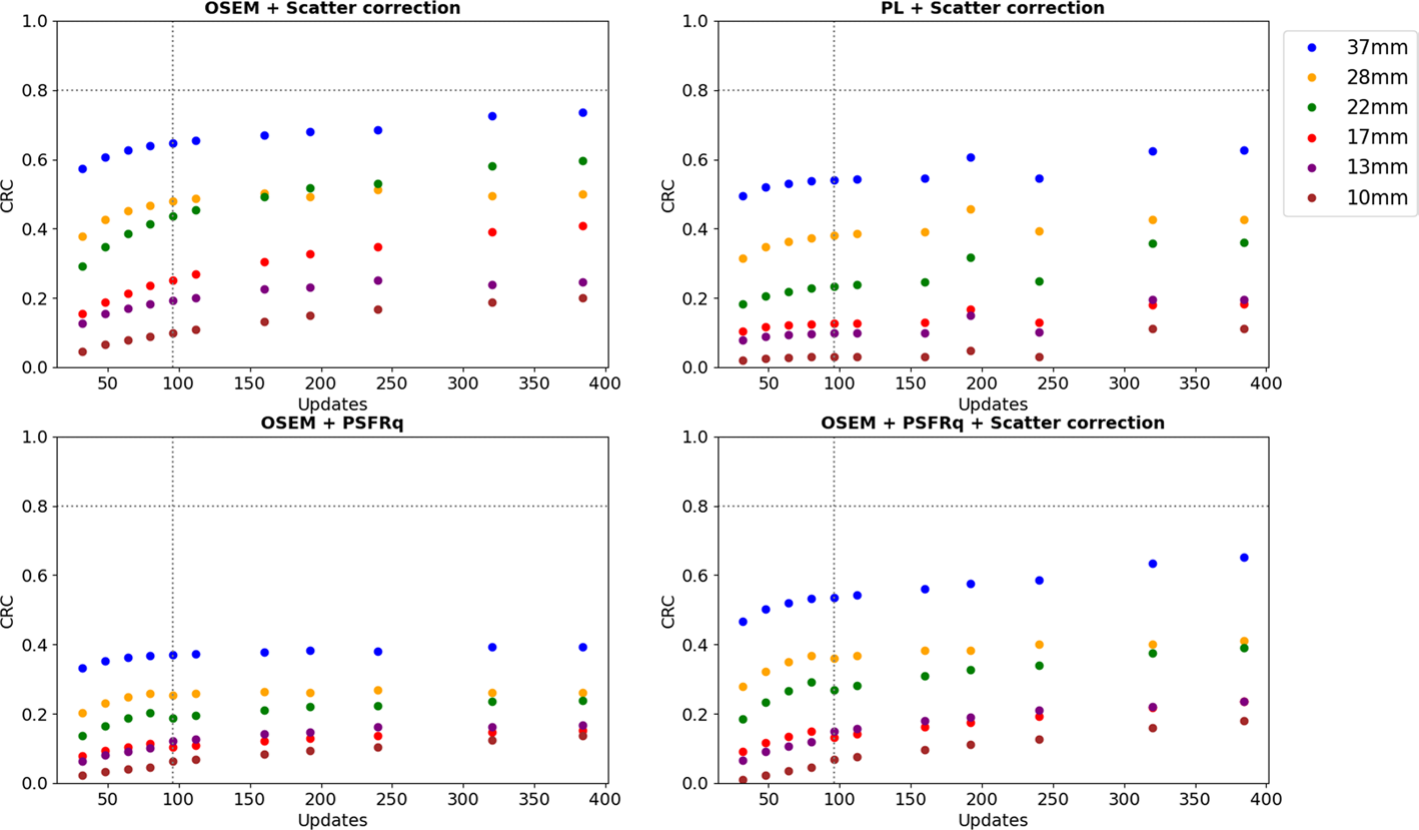
Contrast recovery coefficients (CRC) for different volumes of spheres and vials as a function of the number of updates. At the top left, a scatter correction is applied. Top right, a PL reconstruction is used as well as a scatter correction. Below, the PSFRq option is applied with (right) or without scatter correction (left)

**Fig. 5:**
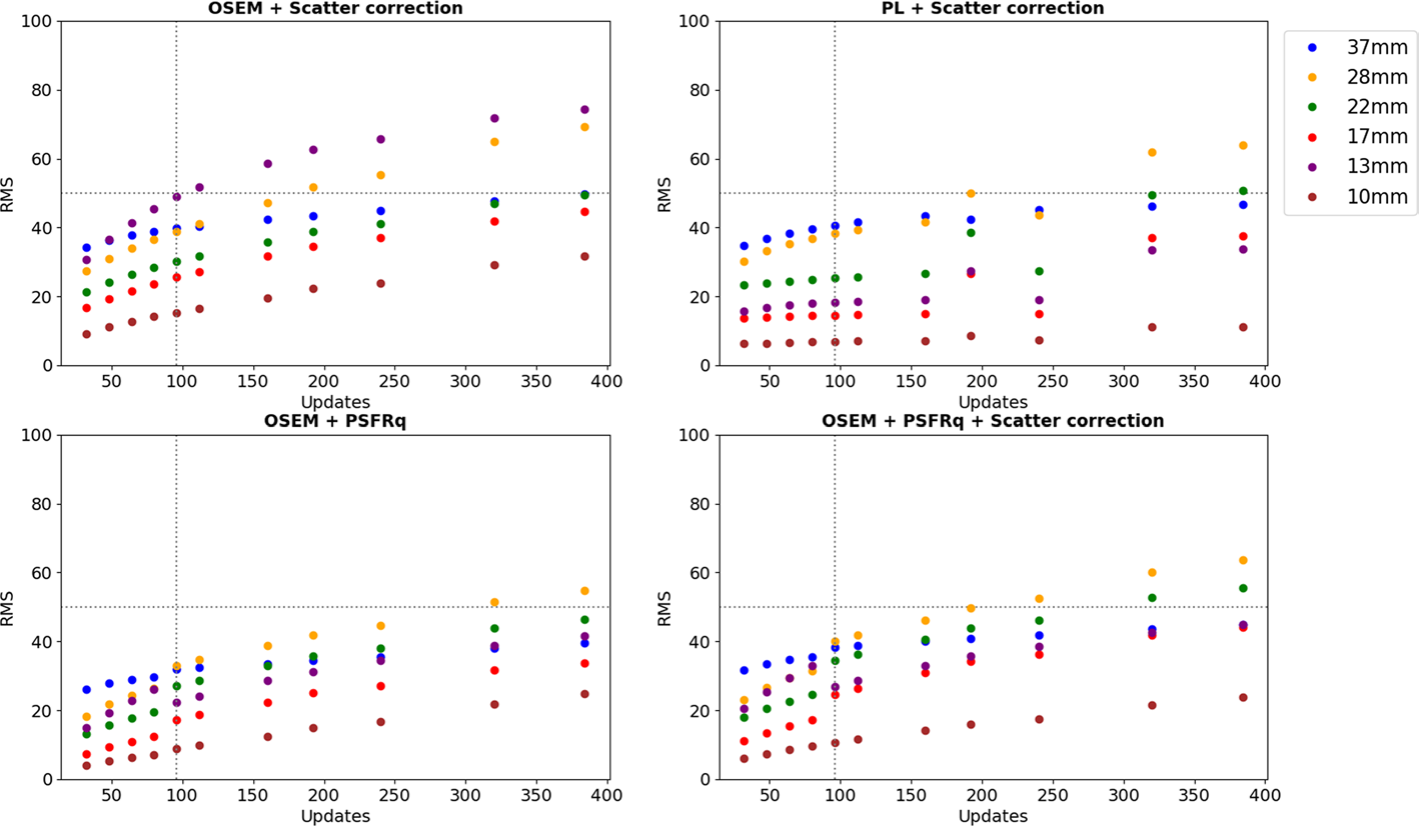
Percentages of root mean square (%) for different volumes of spheres as a function of the number of updates. At the top left, a scatter correction is applied. Top right, a PL reconstruction is used as well as a scatter correction. Below, the PSFRq option is applied with (right) or without scatter correction (left)

Figures 3, 4 and 5 show activity concentration recovery coefficients (ARC), contrast recovery coefficients (CRC) and percentages of root mean square (RMS), respectively, for different numbers of updates and different reconstruction parameters. Background RMS percentages were also evaluated. The calculations were performed using the M1 segmentation method (CT-based contours) using the mean and not the maximum in order to be as close as possible to the patient dosimetry. With increased numbers of updates for OSEM reconstructions with scatter correction and without PSFRq option, the RMS percentage increased from 27–30% for 32 updates to 91–95% for 384 updates whatever the size of the sphere considered. Similarly, the ARC, CRC and RMS percentages increased with the number of updates. Based on these results, a set of optimal parameters was selected. We defined the best compromise by limiting the variability in homogeneous regions (spheres and background) below 50%, which corresponded to 96 updates (12 iterations and 8 subsets). For all subsequent experiments, each reconstruction was performed with OSEM, CT-based attenuation correction, scatter correction, 12 iterations, 8 subsets, no filters, no PSFRq and no regularisation.

### Calibration factors

The calibration factor was evaluated for eight different activity concentrations by using reconstruction parameters determined previously (12 iterations, 8 subsets). This factor was 0.176 ± 0.00444, i.e. a coefficient of variation (CoV) of 2.5% for Ph1.

### Quantification on large volumes

RecQuant was used on phantom Ph3 with hot volumes larger than the spheres, for 5 different concentrations. ARCs were on average 0.91 ± 0.0117 (CoV = 1.3%) for the small “organ” and 0.91 ± 0.0082 (CoV = 0.9%) for the large “organ”.

### Influence of the ROI delineation

**Fig. 6:**
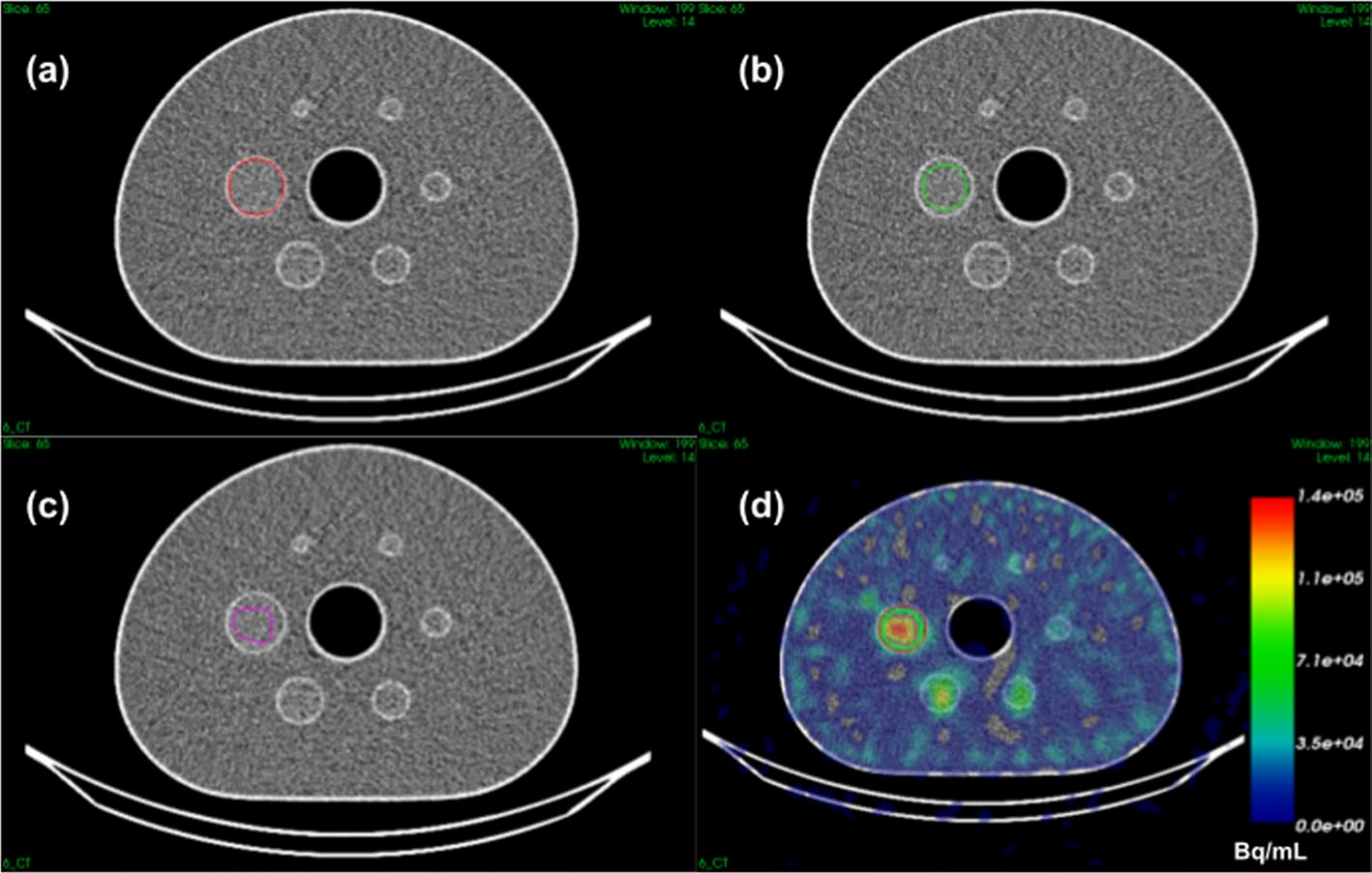
Superposition of the CT and the contour of the 37 mm diameter sphere of the NEMA IEC phantom obtained by **a** anatomical segmentation of the CT (M1), **b** anatomical segmentation of the CT with a margin (M2) or **c** by thresholding the SPECT image (M3). Image **d** is the fusion of the CT and SPECT image (Bq/mL non-calibrated) and the superposition of the contours obtained with the three methods (M1, M2 and M3)

ARCs were estimated for the Ph2 phantom reconstructed with the selected parameters from different segmentation methods (M1, M2 and M3 described in section Material and methods) for average or maximum based figures of merit (Table 5). An example of each segmentation method is given in Fig. 6 for the 37 mm sphere.

**Table 5:** Mean and maximum activity concentration recovery coefficients estimated for each delineation method (M1, M2 and M3). Spheres 1 to 6 have a respective diameter of 37 mm, 28 mm, 22 mm, 17 mm, 13 mm and 10 mm

	Sphere 1	Sphere 2	Sphere 3	Sphere 4	Sphere 5	Sphere 6
M1_*Mean*_	0.67	0.53	0.49	0.33	0.28	0.21
M1_*Max*_	1.37	1.15	0.82	0.49	0.56	0.28
M2_*Mean*_	0.84	0.64	0.60	0.37	0.32	0.18
M2_*Max*_	1.37	1.15	0.82	0.49	0.53	0.23
M3_*Mean*_	0.97	0.81	0.59	0.36	0.39	0.25
M3_*Max*_	1.37	1.15	0.82	0.49	0.56	0.46

### Patient images

**Fig. 7:**
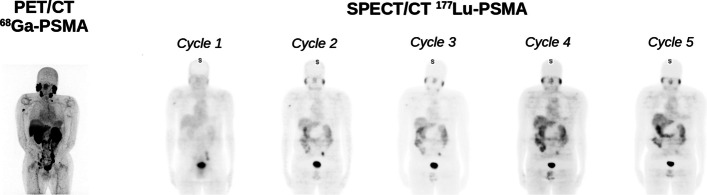
On the left, ^68^ Ga-PSMA PET image for a patient with a metastatic castration-resistant prostate cancer. On the right, 5 planar images obtained from SPECT reconstructions (RecVis) after the first five injections of ^177^Lu-PSMA. The PSA levels measured at each treatment are 63.50, 26.60, 4.51, 1.81 and 0.92 ng/mL, respectively

**Fig. 8:**
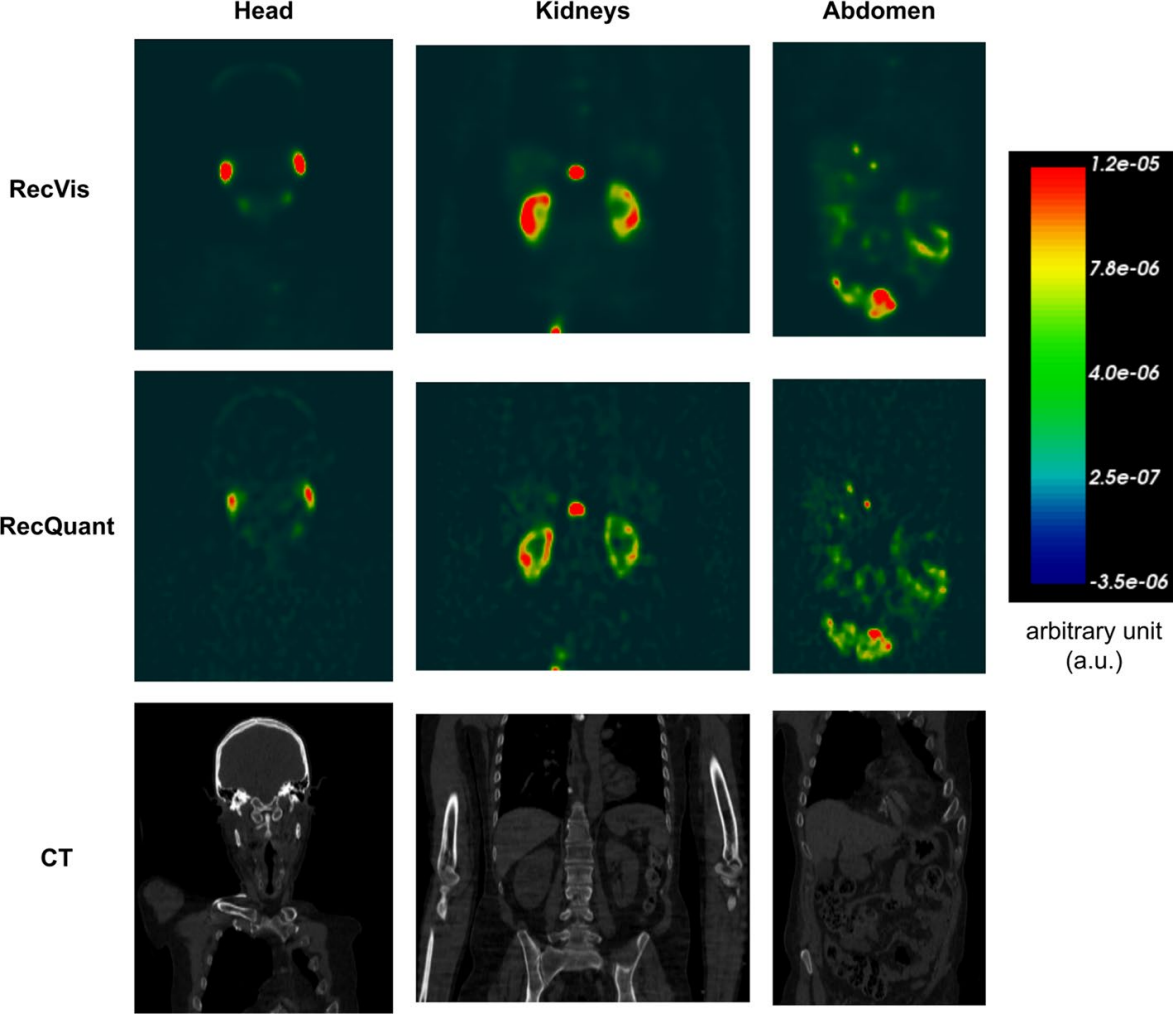
Normalised SPECT reconstructions (RecVis and RecQuant) in arbitrary unit of a patient treated with ^177^Lu-PSMA-1 24H after the first injection. The focus is on three regions: the head, the kidneys and the abdomen. Associated CT scans are also available

Count rates were estimated for all BP, from image acquisitions at three timepoints for three patients. The count rates ranged from 0.1 kcps to 35.3 kcps. Count rates were also evaluated for Ph2 phantom with eight different activity concentrations and ranged from 3.1 kcps to 13.0 kcps. Each BP covers 31.5 cm, with a scan duration of 5 min maximum, reducing to around 2 min/BP for regions without pathological uptake (e.g. legs). A whole-body acquisition (i.e. vertex to toes) requires 6 or 7 BPs, depending on patient size. Hence, total SPECT acquisition duration was between 24 and 26 min overall, including time for detector movements. Typical CT acquisition time is around 1 min. Figure 7 illustrates SPECT and corresponding ^68^ Ga-PSMA PET images for one patient. Five SPECT images were reconstructed using RecVis visualisation parameters and transformed into planar images. Figure 8 shows normalised SPECT images acquired 24H after patient treatment with ^177^Lu-PSMA and reconstructed with RecVis and optimised RecQuant parameters, respectively. For a patient for whom 6 BP have been acquired, the RecQuant reconstruction takes 19 min before scatter correction and 27 min in total when scatter correction is applied.

## Discussion

The objective of this study was to evaluate quantitative performance of the CZT VERITON 360° gamma camera (VERITON-CT 200 series, SW version 2.3.0.1234) and to optimise the reconstruction parameters in the follow-up of patients treated with ^177^Lu-PSMA. Unlike conventional gamma cameras that use a recommended ^177^Lu energy peak at 208 keV (more photons, less scatter) [46], the VERITON system evaluated in this study has a SPECT energy range of 40–200 keV, therefore is only able to recover the 113 keV photopeak (< 200 keV). However, solid-state detectors have better (in the range of 2 to 5% [47]) energy resolution than Anger cameras because it only takes a few eV to create electron–hole pairs [48]. Moreover, CZT has also demonstrated better sensitivity [47] (factor 5 to 10 for myocardial applications). The performance of CZT gamma cameras means that ^177^Lu SPECT images could be improved both qualitatively and quantitatively.

### Determination of optimal reconstruction parameters for quantification

The SPECT image reconstruction was based on iterative OSEM with CT-based attenuation correction [37] and no inter- and/or post-filtering to avoid impacting the quantification [36]. Scatter windows were 22.6 keV width, wide enough to limit noise when using TEW [37]. Reconstruction included a model of the system geometry whose uncertainties are partly corrected with the PSFRq option, which corresponds to a conventional spatially invariant PSF and a correction for blurring effects (e.g. scatter). There is a risk of scatter being corrected twice when the scatter correction is used simultaneously with PSFRq option. For this reason, sets no. 1, no. 3 and no. 4 were tested. For each case, the number of updates was modified in order to determine the best compromise between quantification accuracy and noise. Finally, a fourth case was considered: scatter correction + PL regularisation (case no. 2) which should allow an increase in the number of updates while controlling the noise. For all four sets of parameters, as the number of updates was increased, ARC, CRC and RMS also increased. The minimum number of updates considered corresponds to the default parameters.

These results are consistent with the literature since there is a convergence of ARC and CRC [32] and an increase in noise [38]. In the following, all the intervals given are those of the 37 mm sphere, whatever the number of updates. Where this is not the case, an indication is given. The ARCs were highest for cases no. 1 and no. 4, i.e. with scatter correction, and without/with PSFRq (ARC ∈ [0.63; 0.70]). However, ARCs for the 28 mm sphere were higher without PSFRq (ARC ∈ [0.46; 0.53] whatever the number of updates) than with (ARC ∈ [0.44; 0.50]), whereas the noise was lower with PSFRq option. Without scatter correction, ARCs were low (∈ [0.51; 0.55]) even with PSFRq. Scatter correction is thus essential and recommended for 113 keV, contaminated by downscatter from 208 keV. The use of PL regularisation together with scatter correction led to lower ARCs ([0.57; 0.66] with 0.66 for a number of updates superior to 300) compared to scatter correction alone. Similarly, the CRCs (∈ [0.50; 0.63]) are lower with parameters set no. 2 than with parameter set no. 1 (∈ [0.57; 0.74]). The noise remained low up to 160 updates which shows the benefit of the PL regularisation. The PL regularisation has not been studied in depth because it is still under development by the company Spectrum Dynamics. Set no. 1 (scatter correction only) is thus recommended as it offers the best quantitative results. The recommended number of updates was 96 (12 iterations and 8 subsets) to keep noise below 50%.

In the literature, a few ARC and CRC values are available for CZT imaging systems using ^177^Lu. For example, Kennedy et al.[30] present ARC results for the Discovery 670 CZT for ^177^Lu as a function of the energy peak and collimator chosen. For the 37 mm sphere, for the 113 keV peak with an LE collimator and TEW scatter correction, they obtained an ARC close to 1.2, while for the 208 keV peak, the ARC was 0.6. For the same sphere, the ARC estimated with the set of optimised parameters is 0.7. This can be explained by the difference in geometry and collimator between the systems. A second system designed by the Huh et al. [49] team was evaluated by Monte Carlo simulation. They obtained CRCs of around 0.55 for the 37 mm sphere compared with 0.65 with the optimised parameters of our system.

### Influence of ROI definition

ARC and CRC depend on contours of the spheres. In the literature, several methodologies have been used [32, 41–44] and, among them, three have been studied here (M1, M2 and M3 illustrated in Fig. 6). Moreover, ARC and CRC can be computed with average or maximum concentration within the ROI. This has a major influence on the ARCs, e.g. for the largest sphere using the mean, ARCs were 0.67, 0.84 and 0.97 for M1, M2 and M3, respectively (Table 5). With the maximum, ARCs were identical for most spheres except the smallest ones. Using maximum is noise sensitive and only relies on a single voxel, while using average lowers the ARCs by integrating the heterogeneity of the concentrations within a volume but is less sensitive to noise. Segmentation based on the SPECT image (M3) is dependent on the reconstruction parameters unlike M1 and M2. Here, the mean-based M1 method was chosen because during patient dosimetry, the organs at risk are segmented anatomically on the CT (the only available information) and the mean absorbed dose is calculated within a ROI. In the case of lesions, anatomical contouring can be challenging, making the M1 method unusable. To our knowledge, accurate tumour segmentation is still an open question. In the MIRD no. 23, ARCs superior to 0.9 are considered to be highly accurate reconstructions; however, the methodology associated with these results was not described. In our study, the ARCs reached 0.9 with the M3 method but not with the M1 method.

### Evaluation of the reconstruction parameters

#### Calibration factor

Image acquisitions of the Ph1 calibration phantom were performed at different times in order to vary the activity concentration. The calibration factor was stable with CoV equal to 2.5%. Note that CT contours of the inner edges of the cylinder have been delineated on all images and may be an additional source of uncertainty.

#### Activity recovery in large hot volumes

With volumes close to those of patient organs such as kidneys or spleen (129 and 521 mL), ARCs were greater than 0.9 and therefore suitable for dosimetry. For smaller volumes, additional partial volume correction is advised.

### Patient acquisitions

The RecVis reconstruction shown in Fig. 7 was performed with the parameters recommended by the manufacturer. The HPC option takes account of high-energy photons passing through the collimator; hence, scatter correction is not applied. PSFR option was also applied and has been optimised for visualisation. The acquisition time of a whole-body SPECT scan was reduced by at least half when compared to that of a conventional gamma camera. This is important for patients in pain who may find it difficult to remain still for long periods and where multiple SPECT/CT acquisitions will be performed. In practice, it is also possible to perform more SPECT/CT acquisitions in one day. In our institution, more than 60 patients have already been imaged with this camera for monitoring their treatment. The latest version of VERITON (400 series) allows acquisitions of higher energy photons (up to 400 keV) and thus, the ability to use the peak at 208 keV of ^177^Lu.

## Conclusion

We evaluated the quantitative performance of VERITON for ^177^Lu treatments and proposed a set of recommended reconstruction parameters for quantification purposes called RecQuant. We advocate the use of two sets of reconstruction parameters: one for quantification and one for visualisation with the same acquisition parameters. For quantification, CT-based attenuation correction, scatter correction, 12 iterations, 8 subsets and no filter provided the best compromise between ARC and RMS values. For large volumes such as kidneys, ARCs were in the order of 0.91. In this work, the VERITON 200 series system with SW version 2.3.0.1234 was used. The reconstruction software is still evolving and additional improvements are expected in future versions. While the system is limited to 113 keV detection for ^177^Lu, the ARC obtained with RecQuant demonstrates that it can be used for ^177^Lu dosimetry purpose. The acquisition time for a whole-body image of about 1.8 m length is around 25 min, which is about three times faster than with conventional dual-head cameras.

## Data Availability

Python scripts for activity recovery are available from the corresponding author on request.
